# Supplementary material for: Indocyanine Green-Nexturastat A-PLGA Nanoparticles Combine Photothermal and Epigenetic Therapy for Melanoma
Source: Nanomaterials (Basel). 2020 Jan 17;10(1):161. doi: 10.3390/nano10010161 (PMC7022377; doi:10.3390/nano10010161)
Supplement: Supplementary file 1 [file nanomaterials-10-00161-s001.zip › nanomaterials-676020-supplementary.pdf]

ELECTRONIC SUPPLEMENTARY MATERIAL

Indocyanine Green-Nexturastat A-PLGA Nanoparticles Combine Photothermal and Epigenetic Therapy for Melanoma

Debbie K. Ledezma [1,2], Preethi B. Balakrishnan [2], Juliana Cano-Mejia [2], Elizabeth E. Sweeney [2], Melissa Hadley [2], Catherine M. Bollard [1,2,3], Alejandro Villagra [2], Rohan Fernandes [1,2,4]*

[1] The Institute of Biomedical Sciences, The George Washington University, 2300 Eye Street NW, Room 561, Washington, DC 20037, USA.

[2] The George Washington Cancer Center, The George Washington University, Washington, DC 20052, USA.

[3] Center for Cancer and Immunology Research, Children's National Health System, 111 Michigan Ave NW, Washington, DC 20010, USA

[4] Department of Medicine, The George Washington University, Washington, DC 20052, USA.

*****Correspondence: rfernandes@gwu.edu; Tel.: +1-202-994-0899

This document contains **Supplementary Figures S1-S8 and Tables S1-S3**

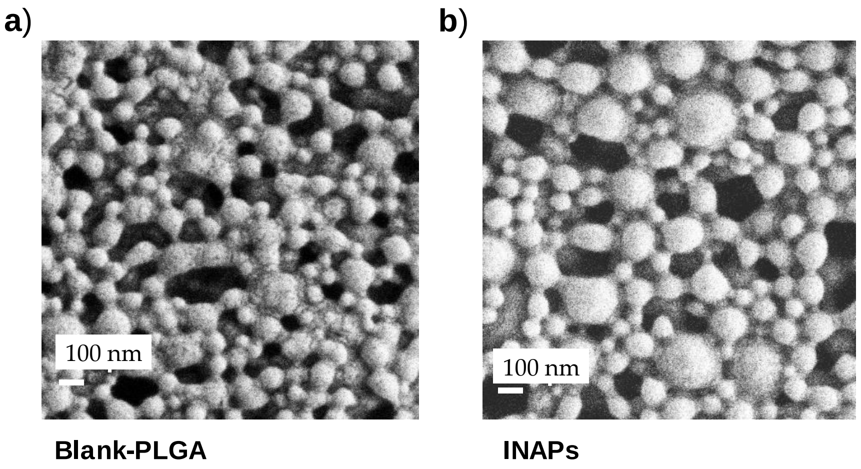

Figure S1. Morphology of Blank-PLGA and INAPs. Scanning electron microscope images of INAPs at high magnification (50,000x, curr 13 pA) for (**a**) Blank-PLGA and (**b**) INAPs.

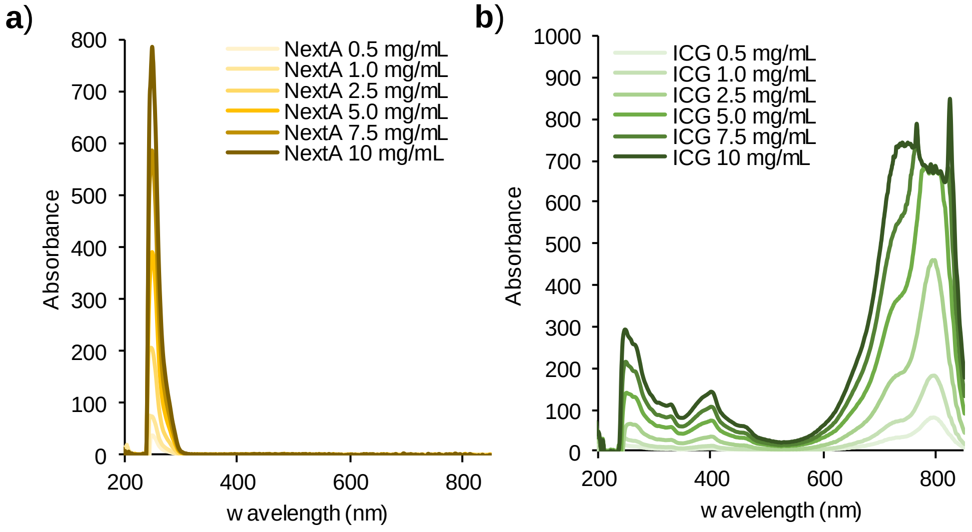

Figure S2. Spectra of free NextA and free ICG at varying concentrations. NextA (**a**) and ICG (**b**) were diluted in DMSO to varying concentrations (0.5 mg/mL- 10 mg/mL). NextA shows a characteristic peak at 250 nm. ICG has a characteristic peak at 780 nm, along with smaller peaks at 256 nm and 400 nm. Spectra was detected using Nanodrop, repeated in triplicates for each concentration.

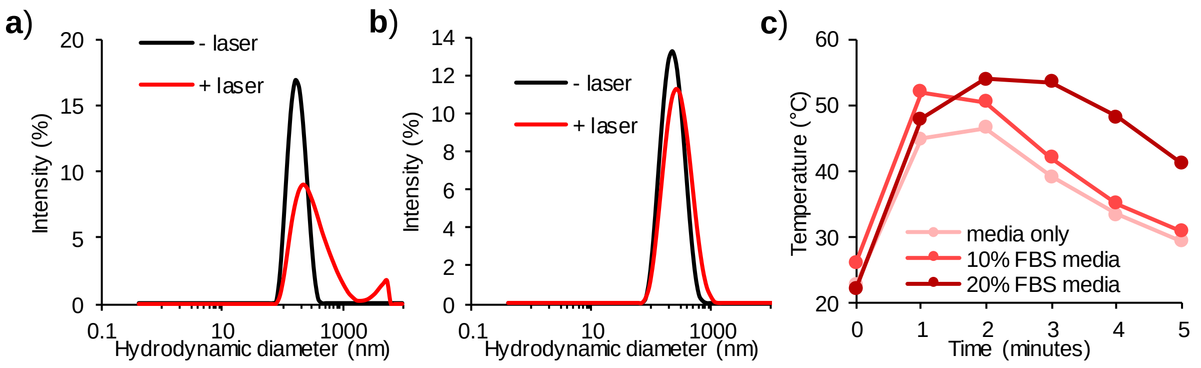

Figure S3. Stability of INAPs upon irradiation with the NIR laser. INAPs (4 mg/mL) were suspended in either (**a**) PBS or (**b**) complete media. The size distributions of the INAPs were assessed before (black) and after (red) NIR laser exposure for 5 minutes at 0.8 W using DLS. (**c**) INAPs (4 mg/mL) were also suspended in media with varying ratios of serum (no serum to 20% FBS) and irradiated with the NIR laser for 5 minutes at 0.8 W. The temperature was measured at 1-minute intervals using the thermal camera.

Table S1. Statistical analysis (p-values) for the study comparing the viability of SM1 cells treated with INAPs, ICG-PLGA, or controls (**a**) before and (**b**) after irradiation with the NIR laser. A two-way ANOVA followed by Sidak's comparison test with multiple comparison's test correction were applied to compare the viability across groups and treatments described in **Figure 3g** in the main text.

a)

Multiple comparison's test (Figure 3g, - laser)	Adjusted P value
Vehicle (water) vs.ICG-PLGA 0.5 mg/mL	0.0022
Vehicle (water) vs.ICG-PLGA 1.0 mg/mL	0.6991
Vehicle (water) vs.ICG-PLGA 2.0 mg/mL	0.8824
Vehicle (water) vs.INAPs 0.5 mg/mL	0.0969
Vehicle (water) vs.INAPs 1.0 mg/mL	>0.9999
Vehicle (water) vs.INAPs 2.0 mg/mL	0.9376
ICG-PLGA 0.5 mg/mL vs. ICG-PLGA 1.0 mg/mL	<0.0001
ICG-PLGA 0.5 mg/mL vs. ICG-PLGA 2.0 mg/mL	0.1764
ICG-PLGA 0.5 mg/mL vs. INAPs 0.5 mg/mL	0.9720
ICG-PLGA 0.5 mg/mL vs. INAPs 1.0 mg/mL	0.0043
ICG-PLGA 0.5 mg/mL vs. INAPs 2.0 mg/mL	<0.0001
ICG-PLGA 1.0 mg/mL vs. ICG-PLGA 2.0 mg/mL	0.0187
ICG-PLGA 1.0 mg/mL vs. INAPs 0.5 mg/mL	0.0005
ICG-PLGA 1.0 mg/mL vs. INAPs 1.0 mg/mL	0.5022
ICG-PLGA 1.0 mg/mL vs. INAPs 2.0 mg/mL	>0.9999
ICG-PLGA 2.0 mg/mL vs. INAPs 0.5 mg/mL	0.9881
ICG-PLGA 2.0 mg/mL vs. INAPs 1.0 mg/mL	0.9697
ICG-PLGA 2.0 mg/mL vs. INAPs 2.0 mg/mL	0.0534
INAPs 0.5 mg/mL vs. INAPs 1.0 mg/mL	0.1719
INAPs 0.5 mg/mL vs. INAPs 2.0 mg/mL	0.0015
INAPs 1.0 mg/mL vs. INAPs 2.0 mg/mL	0.8134

b)

Mulitple Comparison's test (Figure 3g, + laser)	Adjusted P value
Vehicle (water) vs. ICG-PLGA 0.5 mg/mL	0.2936
Vehicle (water) vs. ICG-PLGA 1.0 mg/mL	<0.0001
Vehicle (water) vs. ICG-PLGA 2.0 mg/mL	<0.0001
Vehicle (water) vs. INAPs 0.5 mg/mL	0.0685
Vehicle (water) vs. INAPs 1.0 mg/mL	<0.0001
Vehicle (water) vs. INAPs 2.0 mg/mL	<0.0001
ICG-PLGA 0.5 mg/mL vs. ICG-PLGA 1.0 mg/mL	0.0071
ICG-PLGA 0.5 mg/mL vs. ICG-PLGA 2.0 mg/mL	<0.0001
ICG-PLGA 0.5 mg/mL vs. INAPs 0.5 mg/mL	<0.0001
ICG-PLGA 0.5 mg/mL vs. INAPs 1.0 mg/mL	0.0045
ICG-PLGA 0.5 mg/mL vs. INAPs 2.0 mg/mL	<0.0001
ICG-PLGA 1.0 mg/mL vs. ICG-PLGA 2.0 mg/mL	<0.0001
ICG-PLGA 1.0 mg/mL vs. INAPs 0.5 mg/mL	<0.0001
ICG-PLGA 1.0 mg/mL vs. INAPs 1.0 mg/mL	>0.9999
ICG-PLGA 1.0 mg/mL vs. INAPs 2.0 mg/mL	<0.0001
ICG-PLGA 2.0 mg/mL vs. INAPs 0.5 mg/mL	<0.0001
ICG-PLGA 2.0 mg/mL vs. INAPs 1.0 mg/mL	<0.0001
ICG-PLGA 2.0 mg/mL vs. INAPs 2.0 mg/mL	>0.9999
INAPs 0.5 mg/mL vs. INAPs 1.0 mg/mL	<0.0001
INAPs 0.5 mg/mL vs. INAPs 2.0 mg/mL	<0.0001
INAPs 1.0 mg/mL vs. INAPs 2.0 mg/mL	<0.0001

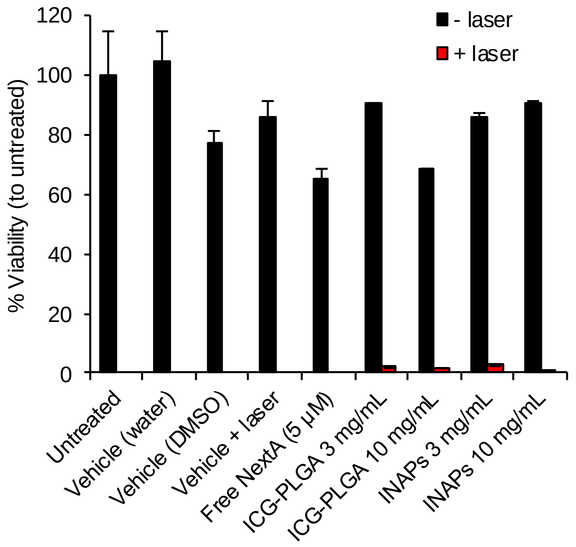

Figure S4. Melanoma cell viability at higher INAPs concentration *in vitro*. B16F10 cells were resuspended in complete RPMI media and treated with INAPs and ICG-PLGA at 3 mg/mL and 10 mg/mL or with controls in the presence (red) or absence (black) of 0.8 W NIR laser for 10 minutes. Treatment with 10 mg/mL INAPs resulted in 0.7% viability compared to untreated control.

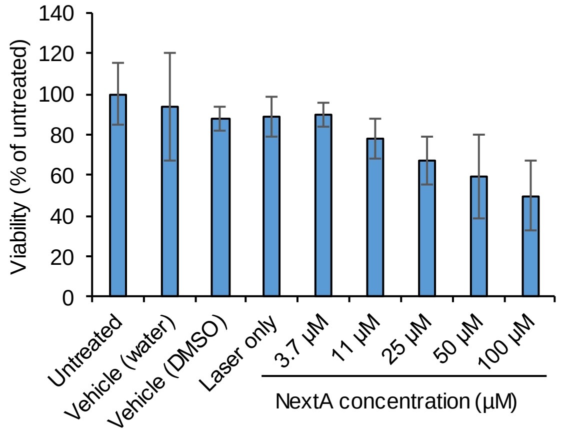

Figure S5. NextA cytotoxicity at varying concentrations. SM1 cells were treated with varying concentrations of NextA (3.7µM – 100 µM) for 24 hours. Cells were collected and resuspended in PBS to determine viability using the Cell Titer-Glo ATP assay. Luminescence for the assay was measured with a SpectraMax plate reader.

Table S2. Statistical analysis (p-values) for the study comparing the inhibition of HDAC activity by INAPs and controls (ICG-PLGA, NextA-PLGA, Free NextA, LBH, and vehicle) across multiple treatments. A one-way ANOVA followed by Sidak's comparison test with multiple comparison's test correction were applied to compare the inhibition of HDAC activity as a function of (**a**) various treatment groups, (**b**) INAP concentration, and (**c**) time as described in **Figure 4** of the main text. Blank-PLGA were omitted from analysis in (**a**).

a)

Multiple comparisons test (Figure 4a, - laser)	Adjusted P Value
Vehicle vs. LBH (5 µM)	0.0001
Vehicle vs. Free-NextA (5 µM)	0.0003
Vehicle vs. NextA-PLGA	0.0001
Vehicle vs. ICG-PLGA	0.0047
Vehicle vs. INAPs	0.0001
LBH (5 µM) vs. Free-NextA (5 µM)	0.9944
LBH (5 µM) vs. NextA-PLGA	>0.9999
LBH (5 µM) vs. ICG-PLGA	0.2223
LBH (5 µM) vs. INAPs	>0.9999
Free-NextA (5 µM) vs. NextA-PLGA	0.9956
Free-NextA (5 µM) vs. ICG-PLGA	0.4434
Free-NextA (5 µM) vs. INAPs	0.9923
NextA-PLGA vs. ICG-PLGA	0.2309
NextA-PLGA vs. INAPs	>0.9999
ICG-PLGA vs. INAPs	0.2105

b)

Multiple comparisons test (figure 4b, - laser)	Adjusted P Value
Untreated vs. Vehicle (DMSO)	>0.9999
Untreated vs. LBH (5 µM)	<0.0001
Untreated vs. Free-NextA (5 µM)	<0.0001
Untreated vs. INAPs 0.5 mg/mL	<0.0001
Untreated vs. INAPs 1.0 mg/mL	<0.0001
Untreated vs. INAPs 2.0 mg/mL	<0.0001
Untreated vs. INAPs 4.0 mg/mL	<0.0001
Vehicle (DMSO) vs. LBH (5 µM)	<0.0001
Vehicle (DMSO) vs. Free-NextA (5 µM)	<0.0001
Vehicle (DMSO) vs. INAPs 0.5 mg/mL	<0.0001
Vehicle (DMSO) vs. INAPs 1.0 mg/mL	<0.0001
Vehicle (DMSO) vs. INAPs 2.0 mg/mL	<0.0001
Vehicle (DMSO) vs. INAPs 4.0 mg/mL	<0.0001
LBH (5 µM) vs. Free-NextA (5 µM)	0.2064
LBH (5 µM) vs. INAPs 0.5 mg/mL)	<0.0001
LBH (5 µM) vs. INAPs 1.0 mg/mL)	<0.0001
LBH (5 µM) vs. INAPs 2.0 mg/mL)	0.0006
LBH (5 µM) vs. INAPs 4.0 mg/mL)	0.2979
Free-NextA (5 µM) vs. INAPs 0.5 mg/mL	<0.0001
Free-NextA (5 µM) vs. INAPs 1.0 mg/mL	<0.0001
Free-NextA (5 µM) vs. INAPs 2.0 mg/mL	0.1027
Free-NextA (5 µM) vs. INAPs 4.0 mg/mL	>0.9999
INAPs 0.5 mg/mL vs. INAPs 1.0 mg/mL	<0.0001
INAPs 0.5 mg/mL vs. INAPs 2.0 mg/mL	<0.0001
INAPs 0.5 mg/mL vs. INAPs 4.0 mg/mL	<0.0001
INAPs 1.0 mg/mL vs. INAPs 2.0 mg/mL	0.0065
INAPs 1.0 mg/mL vs. INAPs 4.0 mg/mL	<0.0001
INAPs 2.0 mg/mL vs. INAPs 4.0 mg/mL	0.0666

c)

**Multiple comparisons test (figure 4c, + laser)**	**Adjusted P Value**
Untreated vs. Vehicle	0.4308
Untreated vs. LBH (5 µM)	<0.0001
Untreated vs. Free-NextA (5 µM)	<0.0001
Untreated vs. Day 0	<0.0001
Untreated vs. Day 7	<0.0001
Vehicle vs. LBH (5 µM)	<0.0001
Vehicle vs. Free-NextA (5 µM)	<0.0001
Vehicle vs. Day 0	<0.0001
Vehicle vs. Day 7	<0.0001
LBH (5 µM) vs. Free-NextA (5 µM)	0.8856
LBH (5 µM) vs. Day 0	0.9766
LBH (5 µM) vs. Day 7	0.9172
Free-NextA (5 µM) vs. Day 0	0.9984
Free-NextA (5 µM) vs. Day 7	>0.9999
Day 0 vs. Day 7	0.9996

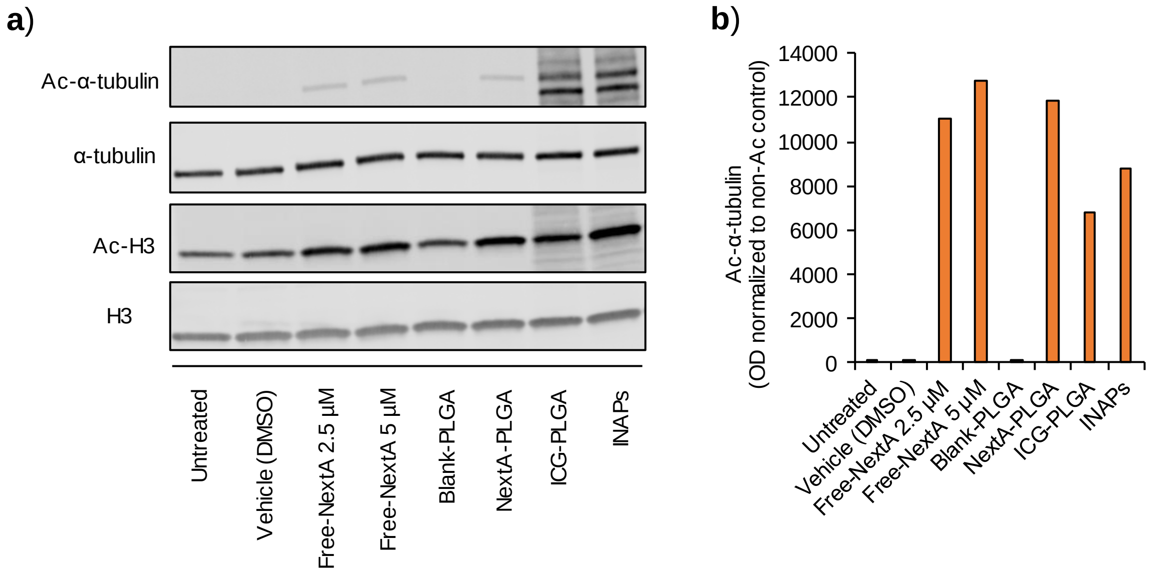

Figure S6. Effect of INAPs on the inhibition of HDAC6 activity in SM1 melanoma cells *in vitro*. SM1 cells were treated with 1.0 mg/mL INAPs for 24 hours. Post-treatment, the cells were collected and lysed for protein collection. (**a**) Cells were blotted for protein expression of Ac-α-tubulin, α-tubulin, Ac-H3, and H3. (**b**) Normalized expression of Ac-α-tubulin. Analysis comparing ICG-PLGA and INAPs was conducted separately from the other groups due to a difference in the background.

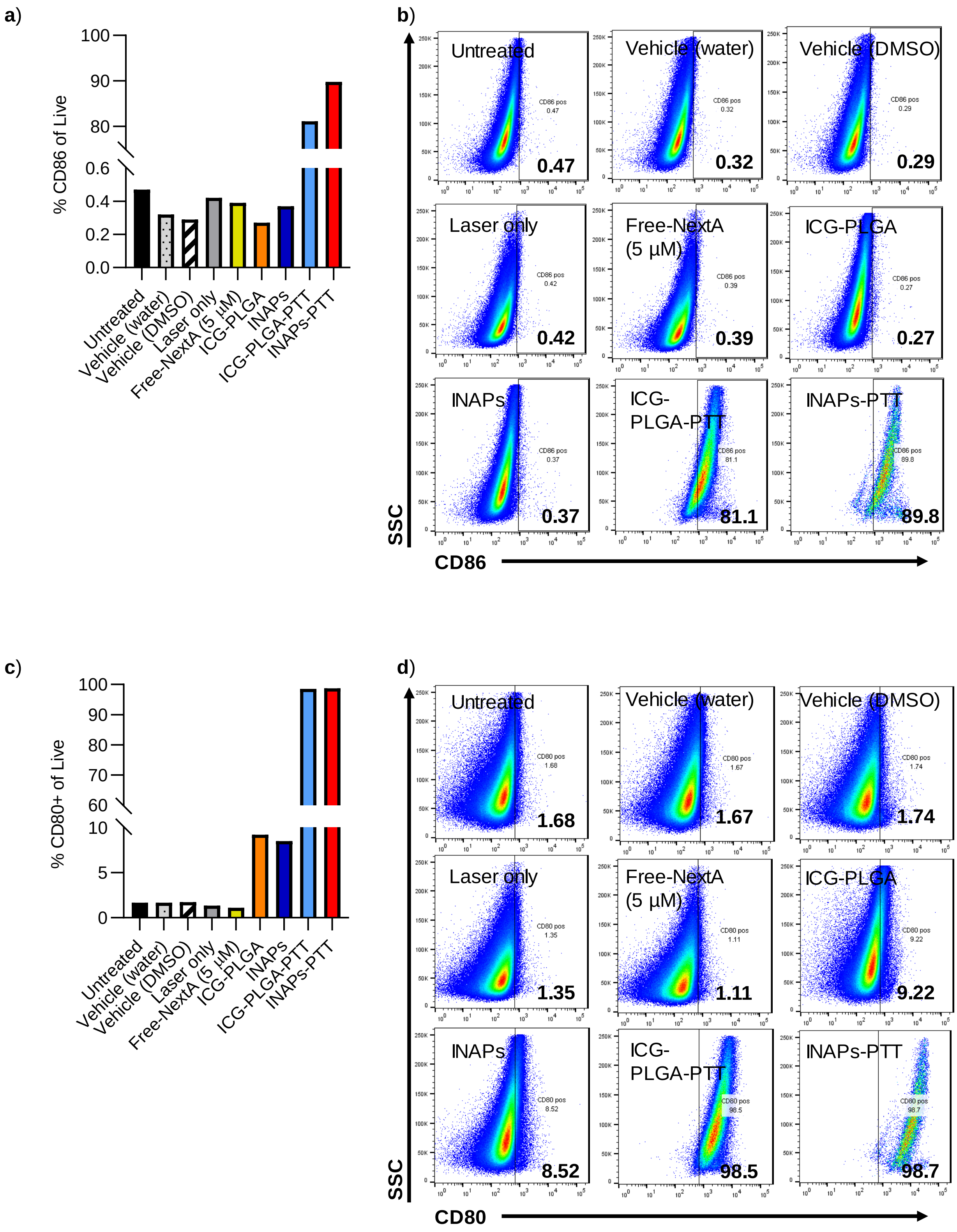
a)
% CD86 of Live
100
90
80
0.6
0.4
0.2
0.0
Untreated
Vehicle (water)
Vehicle (DMSO)
Laser only
Free-NextA (5 µM)
ICG-PLGA
INAPs
ICG-PLGA-PTT
INAPs-PTT
b)
Untreated
0.47
Vehicle (water)
0.32
Vehicle (DMSO)
0.29
Laser only
0.42
Free-NextA (5 µM)
0.39
ICG-PLGA
0.27
INAPs
0.37
ICG-PLGA-PTT
81.1
INAPs-PTT
89.8
SSC
CD86
c)
% CD80+ of Live
100
90
80
70
60
10
5
0
Untreated
Vehicle (water)
Vehicle (DMSO)
Laser only
Free-NextA (5 µM)
ICG-PLGA
INAPs
ICG-PLGA-PTT
INAPs-PTT
d)
Untreated
1.68
Vehicle (water)
1.67
Vehicle (DMSO)
1.74
Laser only
1.35
Free-NextA (5 µM)
1.11
ICG-PLGA
9.22
INAPs
8.52
ICG-PLGA-PTT
98.5
INAPs-PTT
98.7
SSC
CD80

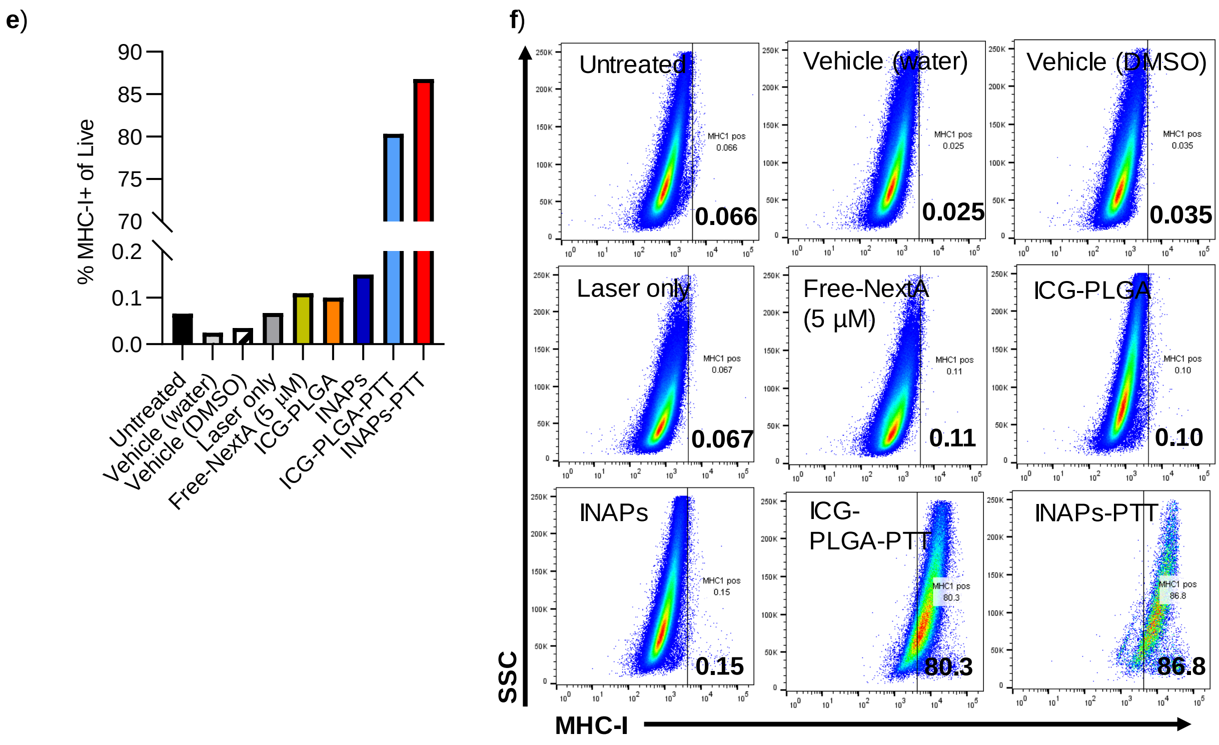

Figure S7. Effect of INAPs-PTT on the expression of immunomodulatory markers on SM1 melanoma cells *in vitro*. SM1 cells were treated with 2.0 mg/mL INAPs and irradiated with NIR laser for 5 minutes at 0.4-0.6 W. Treated cells were incubated overnight and then stained and assessed by flow cytometry for the percentage of live cells expressing of (**a**) CD86, (**c**) CD80, and (**e**) MHC Class I (MHC-I). Respective scatter plots demonstrate gating of (**b**) CD86+, (**d**) CD80+, and (**f**) MHC-I+ live cells. Untreated, water, DMSO, laser only, Free-NextA, and ICG-PLGA served as controls.

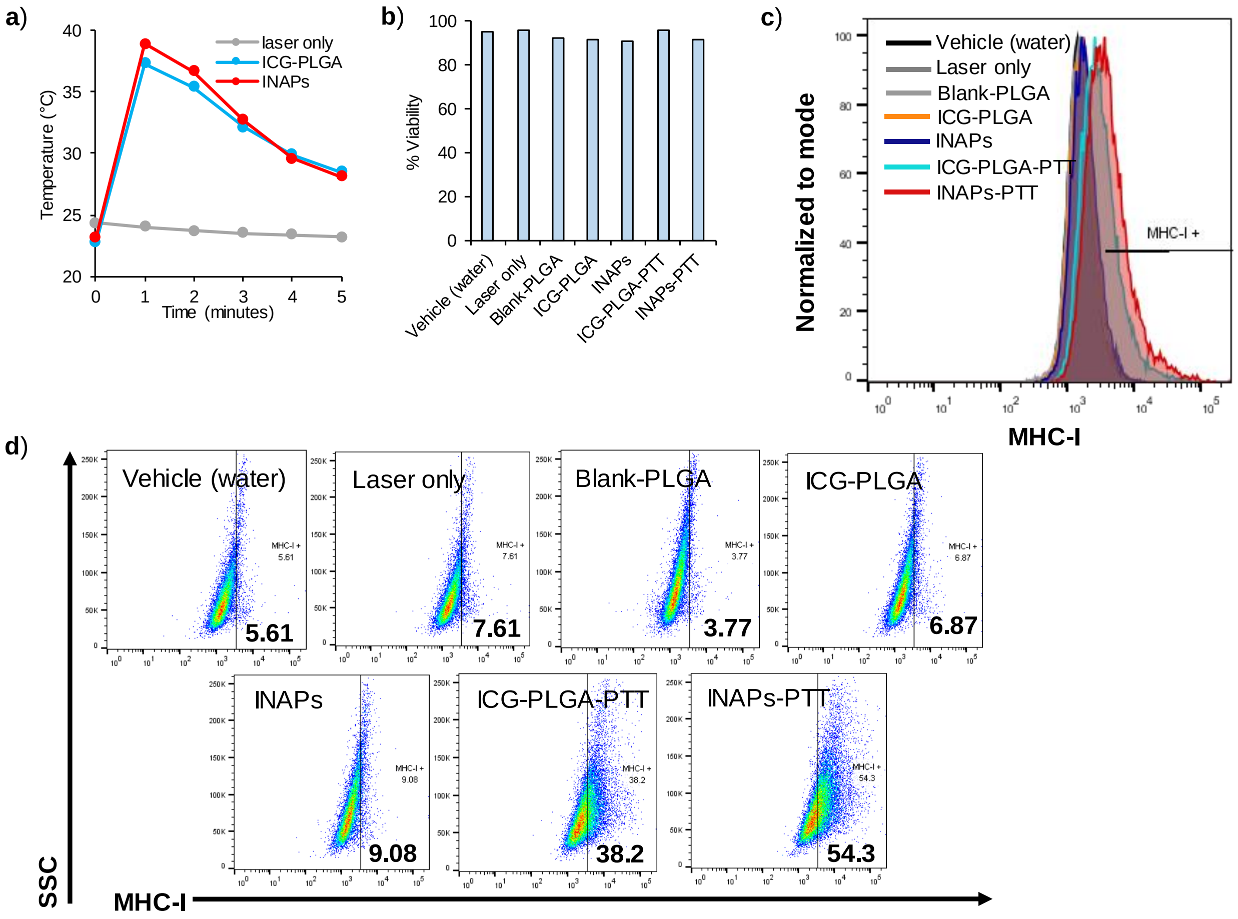

Figure S8. Effect of INAPs-PTT on the expression of immunomodulatory markers on B16-F10 melanoma cells *in vitro*. B16-F10 melanoma cells were treated with 1.0 mg/mL INAPs and irradiated with NIR laser for 5 minutes at 0.8 W. (**a**) Time-temperature trajectories of B16-F10 cells treated with INAPs-PTT or ICG-PLGA-PTT. The treated cells were incubated overnight, stained, and assessed by flow cytometry for (**b**) viability, (**c**) MHC-I expression (MFI), and (**d**) percentages live cells expressing MHC-I.

Table S3. Description of the PLGA nanoparticle properties of the cited references in the Discussion section (**section 4**) of the main text.

a)

**Drug-loaded PLGA nanoparticles compared to free drug**						
**Nanoparticle**	**Cancer/disease**	**Type of synthesis**	**Drug(s) encapsulated**	**Type of therapy**	**Effect compared to free drug(s)**	**Ref.**
5-FU-loaded PLGA nanoparticles	glioblastoma (U87MG), breast cancer (MCF7)	double-emulsion	5-Fluorouracil	chemotherapy	higher S-phase cycle arrest	4
NUC-PLGA nanoparticles	obesity, atherosclerosis (HepG2, Sprague Dawley)	emulsion	Nuciferine	metabolism	lower intracellular triglyceride accumulation; improved pharmacokinetics in plasma	7
DOX-CUR loaded PLGA nanoparticles	leukemia (K562)	single emulsion	Doxorubicin, Curcumin	adjuvant chemotherapy	induce apoptosis at lower concentrations compared to free drug, enhanced K562 cytotoxicity	8
PTX-CP PEG-PLGA nanoparticles	non-small cell lung cancer (344SQ, H460, A549)	Nano-precipitation	Paclitaxel, Cisplatin	chemo-radiotherapy	improved cytotoxicity wit CP-loaded NPs, improve antitumor efficacy of radiotherapy	9

b)

**Co-Encapsulation in PLGA nanoparticles**						
**Nanoparticle**	**Cancer/disease**	**Type of synthesis**	**Drug(s) encapsulated**	**Type of therapy**	**Effect compared to free drug(s)**	**Ref.**
PLGA-ICG-R837 nanoparticles	metastatic cancer (4T1, CT26)	single emulsion	Indocyanine green (ICG), R837	immune-adjuvant photothermal therapy	higher DC maturation and activation, slower secondary tumor growth rate, improved survival with anti-CTLA-4	22
DOX-ICG PEG-PLGA nanoparticles	breast cancer (MCF-7, MCF-7/ADR)	single -step sonication with nanoprecipitation	Doxorubicin, ICG	chemo-photothermal therapy	enhanced cellular uptake of drugs, synergistically induced apoptosis and cell death of chemo-resistant cancer cells, higher drug accumulation in tumor, improved survival	42
